# Gender norms and family planning amongst pastoralists in Kenya: a qualitative study in Wajir and Mandera

**DOI:** 10.1080/26410397.2022.2135736

**Published:** 2022-11-23

**Authors:** Leah Kenny, Michelle Lokot, Amiya Bhatia, Rahma Hassan, Shannon Pyror, Nana Apenem Dagadu, Abdullahi Aden, Abdalla Shariff, Loraine J. Bacchus, Mazeda Hossain, Beniamino Cislaghi

**Affiliations:** a Research Officer, London School of Hygiene and Tropical Medicine, London, UK.; b London School of Economics and Political Science, London, UK; c Assistant Professor, London School of Hygiene and Tropical Medicine, London, UK; d PhD Fellow, University of Nairobi, Nairobi, Kenya; e Family Planning Technical Lead, Save the Children, Washington DC, USA; f M&E Advisor, Reproductive Health, Save the Children, Washington DC, USA; g Programme Manager, Wajir Field Office, Save the Children, Nairobi, Kenya; h Programme Manager, Mandera Field Office Save the Children, Nairobi, Kenya; i Associate Professor, London School of Hygiene and Tropical Medicine, London, UK; j Associate Professorial Research Fellow, London School of Hygiene and Tropical Medicine, London, UK; k Honorary Associate Professor, London School of Economics and Political Science, London, UK

**Keywords:** family planning, social norms, gender norms, pastoralist, semi-nomadic, nomadic, social and behaviour change

## Abstract

There is growing recognition among global health practitioners of the importance of rights-based family planning (FP) programming that addresses inequities. Despite Kenya achieving its national FP target, inequities in access and use of modern FP remain, especially amongst marginalised nomadic and semi-nomadic pastoralist communities. Few studies explore norms affecting FP practices amongst nomadic and semi-nomadic pastoralists and how these can influence social and behaviour change (SBC) interventions. We carried out 48 in-depth interviews and 16 focus group discussions with women and men from pastoralist communities in North Eastern Kenya in November 2018. Data were analysed thematically. Results from focus groups and interviews confirmed themes, while allowing differences between the qualitative approaches to emerge. We found that large family size was a descriptive and injunctive norm in both nomadic and semi-nomadic communities. The desire for around 10 children was sustained by religious beliefs and pastoralist ways of living. Despite a desire for large families, maintaining child spacing was encouraged and practised through breastfeeding and sexual abstinence. Most participants viewed modern FP negatively and as something used by “others”. However, it was acceptable in order to prevent severe negative health outcomes. Future FP research to inform interventions should continue to consider community fertility preferences and the rationale for these, including norms, religion and power dynamics. Targeted qualitative social norms research could inform multi-component SBC interventions in this context.

## Résumé

Les praticiens de santé dans le monde sont de plus en plus conscients de l’importance d’une programmation de la planification familiale fondée sur les droits pour s’attaquer aux inégalités. Même si le Kenya a atteint son objectif national de planification familiale, des inégalités demeurent dans l’accès et l’utilisation de méthodes modernes de contraception, en particulier parmi les communautés pastorales nomades et semi-nomades. Peu d’études examinent les normes qui affectent les pratiques de planification familiale parmi les populations pastorales nomades et semi-nomades et la manière dont elles peuvent influencer les interventions de changement social et comportemental. En novembre 2018, nous avons mené 48 entretiens approfondis et 16 discussions de groupe avec des femmes et des hommes issus de communautés pastorales du nord-est du Kenya. Les données ont été analysées thématiquement. Les résultats des groupes de discussion et des entretiens ont confirmé les thèmes tout en permettant aux différences entre les approches d’apparaître. Nous avons constaté que la famille nombreuse était une norme descriptive et injonctive dans les communautés nomades aussi bien que semi-nomades. Le désir d’avoir une dizaine d’enfants était soutenu par les croyances religieuses et les modes de vie pastoraux. En dépit du souhait d’avoir de nombreux enfants, l’espacement des naissances était encouragé et pratiqué par l’allaitement maternel et l’abstinence sexuelle. La plupart des participants avaient une conception négative des méthodes modernes de contraception et considéraient que c’était quelque chose que « d’autres » utilisaient. Néanmoins, ces méthodes étaient acceptables pour prévenir de graves problèmes de santé. Les futures recherches sur la planification familiale pour guider ces interventions devraient continuer à tenir compte des préférences communautaires en matière de fécondité et des motifs les étayant, notamment les normes, les croyances religieuses et la dynamique de pouvoir. Une recherche qualitative ciblée sur les normes sociales pourrait informer les interventions de changement social et comportemental à plusieurs composantes dans ce contexte.

## Resumen

A nivel mundial, los profesionales de salud cada vez más reconocen la importancia de los programas de planificación familiar (PF) basados en los derechos que abordan las desigualdades. A pesar de que Kenia logró su meta de PF nacional, aún existen desigualdades en la accesibilidad y el uso de la PF moderna, especialmente en las comunidades nómadas y seminómadas pastoralistas marginadas. Pocos estudios exploran las normas que afectan las prácticas de PF entre las comunidades pastoralistas nómadas y seminómadas, y cómo esas normas pueden influir en las intervenciones de cambio social y de comportamiento. Realizamos 48 entrevistas a profundidad y 16 discusiones en grupos focales con mujeres y hombres de comunidades pastoralistas en el noreste de Kenia, en noviembre de 2018. Se analizaron los datos temáticamente. Los resultados de los grupos focales y las entrevistas confirmaron los temas, a la vez que permitieron que surgieran diferencias entre los enfoques cualitativos. Encontramos que tener una familia numerosa era una norma descriptiva y cautelar tanto en las comunidades nómadas como en las seminómadas. El deseo de tener unos 10 hijos era apoyado por creencias religiosas y formas de vivir pastoralistas. Pese al deseo de tener familias numerosas, mantener el espaciamiento de hijos era fomentado y practicado por medio de la lactancia materna y la abstinencia sexual. La mayoría de los participantes tenían un concepto negativo de la PF moderna como algo utilizado por “otros”. Sin embargo, era aceptable a fin de prevenir graves resultados de salud negativos. Futuras investigaciones de PF para informar las intervenciones deben continuar considerando las preferencias de fertilidad de la comunidad y sus fundamentos, tales como normas, religión y dinámica de poder. Las investigaciones cualitativas focalizadas en normas sociales podrían informar intervenciones sobre el cambio social y de comportamiento con múltiples componentes en este contexto.

## Background

Through the FP2020/FP2030 movement, global and national organisations recommitted to expanding equitable access to modern family planning (FP) methods that allow women and couples to delay, limit and space the number of children they have.^1^ These commitments focus on equity and the possibility to transform social and gender norms through social and behaviour change (SBC) strategies, alongside investments in strong health systems, programmes and supply chains. During FP2020 in 2012, Kenya committed to a target of 58% modern FP use by married women, which has since been achieved.^2^ National inequities in access to, and use of, modern FP persist however,^3^ especially amongst marginalised and disadvantaged populations, including nomadic and semi-nomadic communities.

Wajir and Mandera counties, located in the fragile and precarious arid and semi-arid lands of North Eastern Kenya, represent some of the most marginalised counties in Kenya. Though accurate census data are missing,^4^ the majority of the population are nomadic or semi-nomadic pastoralists who are ethnically Somali and religiously Muslim. Nomadic pastoralists rear livestock and migrate seasonally in search of pasture and water.^5^ Structural inequalities, including poor roads, limited communication and health infrastructure, limited funding and poor provision of public services, as well as few national policies that meet nomadic pastoralist health needs, have resulted in Wajir and Mandera having poorer health outcomes than the national average.^6,7^ Solutions such as mobile clinics and the integration of animal and human health services have been proposed,^8,9^ but are yet to be actioned. Despite a national policy in Kenya that promotes FP, the specific sexual and reproductive needs of nomadic and semi-nomadic groups are not adequately addressed (e.g. strategies have not been contextualised for mobile populations). As such, the most recent and available data for Wajir and Mandera counties indicate a very low modern contraceptive prevalence rate, for married women of reproductive age (mCPR, 2.3% and 1.9% respectively) with a high total fertility rate (TFR 7.8 and 5.2, respectively).^3^

Globally, there exist multiple barriers to FP use^10–12^ often determined by contextual factors.^13^ Reasons for non-use of FP are situated across domains at the individual, social, and institutional levels.^14^ The role of social and gender norms in global FP programming has been acknowledged, including how these intersect across various domains of influence.^15^ Social norms are the unwritten rules regulating acceptable actions in a given group^16^ and play an important role in the uptake of FP methods and in defining (un)acceptable birth intervals.^17–20^ The literature on social norms distinguishes between two types that govern individual and collective behaviour: norms that describe the behaviour of other community members (descriptive norms) and norms that set out the level of anticipated disapproval or approval of a certain action (injunctive norms).^21^ Similarly, gender norms that define a woman’s and a man’s role in the couple, family, and society, impact power relations that in turn can limit women’s use or access to health services and any form of FP.^22^ Norms can be classified based on their levels of influence, from weak to strong, which impact health behaviours alongside other individual, social, structural, and material factors.^23^

Literature from other pastoralist communities within the region indicates the importance of norms in influencing uptake of FP. Patriarchal gender norms can play a role in determining decision-making about FP and child spacing.^24^ Studies focused on men’s involvement in decision-making around FP are limited among pastoralist communities. However, in Ethiopia, one study found that most families made joint decisions about FP. Almost half the women in that study indicated a history of their husband objecting to FP use^25^ and others have shown the key role husbands play in shaping women’s FP use.^26^ Social and cultural norms can also influence how pastoralist communities view interventions related to FP.^27^ For example, in the Karamoja region of Uganda, the number of children a woman produces is tied to her status.^28^ Alongside norms, the belief that children are from God is important in shaping FP use among pastoralist communities.^29^

The focus on SBC in designing interventions relates to the systematic application of interactive processes and strategies, based on theory and research, to address SBC at the individual, family, and community levels, including social and gender norms (Save the Children Definition, adapted from C-Change Modules). SBC interventions that seek to shift health-related behaviours have been successful in reducing harm and improving health across a range of public health areas including gender-based violence,^30^ infectious disease,^31^ and sexual and reproductive health,^14^ including family planning,^32^ as well as reducing harmful practices.^33^ SBC interventions draw on the socio-ecological model for change, to identify several levels of influence on the uptake or maintenance of behaviours addressing a variety of primary audiences and their reference groups at the individual, interpersonal, community, and structural level.^34,35^ Careful consideration needs to be given to which norms are barriers to behaviour change in order to be culturally appropriate and effective. Norm change interventions can have inadvertent consequences for some community members, especially where existing gender structures and power dynamics are challenged.^22^

In the context of FP interventions, contraceptive programmes in the global South are largely funded by the global North. As such, a sensitive and nuanced application of an SBC framework is always needed, to achieve SBC’s aim of being culturally appropriate and for programmes to be most effective.^36,37^ An awareness of whose voices are included in SBC intervention design and in the review of unequal power dynamics, not only at community level but also between programme designers in the global North and global South, is crucial for effective and sustainable programme design.^38^ Despite emphasis on reproductive choice and empowerment, FP programmes can take multiple coercive forms and current contraceptive measures do not consistently account for quality of services or choice.^39–41^ Programmes that assume a demand or outcome, may fail to meet need. For example, in North Eastern Kenya unmet need for *spacing* is high, yet the unmet need for *limiting* children is low.^42^ This highlights the importance of engaging with communities to explore preferences and inform interventions, which has increasingly become an important part of FP research and SCB intervention design.^35,43^

The Nomadic Health Project, a four-year partnership between Save the Children, the London School of Hygiene and Tropical Medicine and Centre for Behaviour Change Communication (December 2017–April 2022), seeks to increase access to quality FP services amongst nomadic and semi-nomadic pastoralists in North Eastern Kenya (Wajir and Mandera). The first stage of the project involved formative research and mapping of social and gender norms, the results of which are presented in this paper. This formative research, a review of health system barriers to FP and project stakeholder planning meetings, informed the development of an SBC strategy. The strategy was designed for nomadic and semi-nomadic communities. It aims to increase knowledge around FP and shift norms that prevent women and couples accessing FP in this context, through various channels (at individual, couple, community, system, and policy levels).^44^

Our prior formative qualitative research in North Eastern Kenya highlights how gendered norms influence early and child marriage practices amongst pastoralists and associated early childbearing,^45^ as well as men’s role in FP decision-making^46^ and possible health provider bias in providing FP services in this context.^47^ Few studies explore the reproductive health preferences, including fertility desires and contraceptive preferences, of nomadic and semi-nomadic pastoralist women and men, or the social and gender norms underpinning these in Kenya. Where qualitative studies exist, they point to important socio-cultural factors around family size.^48^ One study from Lamu and Wajir counties noted that religious beliefs, in particular, explained large family sizes.^49^ In this paper, we analysed data from both in-depth interviews (IDIs) and focus group discussions (FGDs) with nomadic and semi-nomadic women and men of different ages, living in North Eastern Kenya. Drawing on our formative qualitative research, we explore: (1) gender and social norms around child spacing and modern FP, (2) family size preferences, and (3) how these approaches to spacing relate to local and global approaches to FP. Both FGDs and IDIs explored these topics, however, IDIs allowed us to gather more detailed information on participants and their child spacing preferences, while FGDs focused more on social norms around family size. Using both methods, we compare findings to see how they differ. Finally, we present implications for SBC intervention design amongst nomadic and semi-nomadic pastoralists in North Eastern Kenya and similar settings.

## Methods

### Study sites and participants

Participants were from Wajir and Mandera counties, situated on the Kenyan side of the Somalia-Kenya border, ethnically Somali, and Muslim. Participants in this study were either nomadic or semi-nomadic pastoralists. Nomadic and semi-nomadic communities differ in their level of sedentarism (e.g. semi-nomadic pastoralists settle for longer periods of time and closer to towns). However, both community types migrate with their livestock (predominantly camel and cattle), or as a result of inter-communal conflict.

We interviewed women and men from one nomadic and one semi-nomadic community in Wajir (Tarbaj and Wajir North sub-counties, respectively) and one nomadic and one semi-nomadic community in Mandera (Mandera North and Takaba sub-counties, respectively). Study sites were identified by Save the Children, working in the area. To identify study participants, male community leaders (chiefs and community elders) were approached to find women and men of reproductive age (15–49 years) for both the FGDs and IDIs. We conducted eight FGDs with younger (18–25 years) and eight with adult (26–49 years) groups. For the IDIs, we sampled participants to represent a range of age groups: adolescents (15–18 years), middle-age (19–35 years), and older (36–49 years). We also interviewed unmarried individuals in the younger age groups, to explore if norms differed among the younger generation (*n* = 8). It was expected that younger, unmarried participants would not be sexually active. We present quotes with details of the participant’s age; the grouping varies depending on whether they took part in an FGD or an IDI. We also present participant numbers (for the FGDs) and FGD and IDI identification numbers for clarity.

### Study design and tools

All interviews were conducted in November 2018. Six interviewers (three men and three women) who were from Wajir and Mandera, were selected by RH as they had experience conducting qualitative interviews in other settings in Kenya and spoke Borana, Somali, and English. Prior to data collection, interviewers participated in a one-week training on the ethics of conducting qualitative interviews, including on sensitive topics. During this period, interview guides were also piloted, and translations finalised. IDIs and FGDs were carried out in parallel, reflecting the equal importance attributed to each data collection method.

Due to the sensitive nature of discussing reproductive and sexual health-related issues, FGDs were stratified by gender and age.^50^ FGD guides were semi-structured, and we used interactive activities to open discussions around typical family size, desired family size, and barriers and facilitators to achieving these family sizes. For example, during a family size activity, participants were asked to place objects beside images representing the ideal number of children for women in their community, and then asked to place objects beside the number representing how many children women were having. The younger groups were also asked about the ideal number of children question, but the second question asked how many children they *expected* to have over the course of their lifetimes (Table 1). Participants were asked to agree as a group on their desired number of children. This activity was used by facilitators to discuss discrepancies between actual and desired family sizes, and the acceptability of different family sizes. The FGD concluded with a vignette about a couple using modern FP, to elicit norms (see Box 1).

**Table 1.** FGD participants number of children agreed on during discussion

Community type	Age group^a^	Gender	Ideal number of children agreed on in the FGD^b^	Number of children achieved by women in FGD participants’ community, median^c^
Nomadic	Younger	Female	8	6.5
			13	8.5
		Male	15	15+
			15+	15
	Adult	Female	15	15
			13	9
		Male	9	9
			12	8
Semi-nomadic	Younger	Female	15	5.5
			12	11
		Male	9	15+
			10	15+
	Adult	Female	14	7.5
			12	13
		Male	12	9
			10	10

a. Younger age group: 18–25 years. Older age group: 26–49 years.

b. Number agreed on by FGD participants as the ideal number of children for a woman in their community, following group exercise.

c. Presented as a median of participant answers. Younger groups were asked how many children they expected they would have over their lifetime. Adult groups were asked how many children the women in their community had on average.

**Box 1.** Example excerpt of vignette used in focus group discussions

Interview type	Vignette (excerpt)
Focus group discussion	This is the story of Saadia and Hassan. They are both 28 years old. Saadia and Hassan have been married for 10 years, they have four children, two girls and two boys. Saadia is Hassan’s only wife, as Hassan couldn’t afford having more. In the last few months Saadia has been very tired with having to take care of the children, as well as her husband. She really feels that she would like to wait to have their next child for a while now, maybe for a couple of years. 1. Is there anything that Saadia could do to delay her next pregnancy? 2. Whose help could Saadia seek? 3. Would most women in Saadia’s situation speak with her husbands about this? Why/why not?

Semi-structured individual interview guides covered four main areas: family formation; family structure and family size; FP methods; and the impact of conflict on fertility and reproductive health. IDIs explored social norms surrounding family size and FP, using vignettes similar to those used in FGDs. Questions explored typical family sizes in the communities (for example, “Thinking about your community, how many children in total do women have on average in the course of their life?”) and participant opinions and explanations for this. Questions around FP asked about child spacing practices and communication around this, knowledge and acceptability of modern methods, education around, and access to, modern FP. For this paper, we focus on family size and family planning questions.

### Data collection

All interviews and FGDs were conducted in the villages where participants were identified and currently lived. Interviewers located a quiet and confidential space for these to take place, away from the central area of the village. FGDs were conducted separately for men and women and each FGD lasted around 60 minutes. FGDs were facilitated by two same-sex moderators with groups of between 6 and 12 individuals. Semi-structured IDIs were carried out with 48 individuals (24 women, 24 men) and were conducted by same-sex interviewers. Demographic information (including age, age married, and number of children) was also collected at the time of interview.

All participants received background information on the research project and provided oral consent to participate and to be audio recorded. Participants were informed they could stop the interview at any point. This study received ethical approval from the London School of Hygiene & Tropical Medicine (ref: 16109) and from Amref in Kenya (ref: AMREF-ESRC P542/2018) in October 2018.

### Data analysis

Interviews were transcribed and translated into English by native Somali and Borana speakers. To ensure data quality, a handful of transcripts were randomly checked by a member of the research team fluent in the local languages and English.

The conceptual framework for this study was developed by MH and BC and acknowledges how social and gender norms intersect at the individual, social, material, institutional, and global level to influence family formation, family structure (e.g. family size), and FP methods, in turn influencing FP use.^51^ Initially, authors RH and LK conducted thematic analysis across all IDIs and FGDs and read through the transcripts in their entirety. They then individually coded the IDIs in Nvivo 12. The authors maintained a shared comment book to review when they cross checked and created the final codebook. The codebook was updated six times, and the final version contained 43 codes. The authors then read through the FGDs and checked codes from IDIs where relevant. The final FGD codebook overlapped with the IDI codebook, with a few exceptions: in FGDs there was more emphasis on desired family size (and the barriers and facilitators to this) and the codebook was shorter (36 codes).

For this paper, authors LK, ML, and AB read selected key codes from the IDI data relating to modern FP, including reference to child spacing, and searched for key themes in these. The codes included: timing of first child; ideal number of children; not ideal number of children; time child spacing; couple discuss child spacing; modern methods of FP; natural methods of FP; and modern FP decision makers. The authors then read through the relevant FGD data, and themes that emerged often and were present in both interview types were included. The two overarching themes included were: desired family size and fertility preferences and acceptable child spacing methods. For all the themes included, we examined how social and gender norms within these themes were discussed by participants (presented in Table 2) and we explored any key tension points in the data which were then included in the final write up.

**Table 2.** Norms identified within the broader themes presented in this paper

Overarching theme	Norms identified across FGD and IDIs	Normative influence
Desired family size and fertility preferences	Women (and men) must give birth to many children	Obligatory^a^/Appropriate^b^ (strong)
	Women must marry young and begin childbearing early^c^	Obligatory (strong)
	Women must give birth to a son	Appropriate (strong)
Acceptable methods of child spacing	Women must space for 2–3 years	Appropriate (strong)
	Women must not use modern methods of family planning	Obligatory (strong)
Gender norms	Men must make reproductive decisions	Obligatory (strong)

a. Describes the level of influence a norm has on an individual, individuals must comply or they face sanctions. All norms in this study were identified as having a strong effect.

b. Describes the level of influence a norm has on an individual, individuals are likely to comply and align their behaviour with what they see around them. All norms in this study were identified as having a strong effect.

c. An in-depth exploration of these gender norms is not presented here as these findings can be found in our paper on child marriage, from this same study.

## Results

Overall, 170 nomadic and semi-nomadic pastoralist women and men participated in our research, through either FGDs (*n* = 16, a total of 122 participants) (Table 3) or in-depth interviews (IDIs, *n* = 48) (Table 4). This included 81 (48%) women and 89 (52%) men. Half of participants had between 6 and 10 children; some men had more than this as they can have multiple wives. Nearly all participants were married (83%) and half had been married between the ages of 15 and 18; however, this was higher amongst women than men.

**Table 3.** Overview of participants in focus group discussions

Community type	Age group^a^	Gender	County	Number of participants
Nomadic (8 FGDs; 57 participants)	Younger (4 FGDs; 31 participants)	Female	Wajir	8
			Mandera	6
		Male	Wajir	10
			Mandera	7
	Adult (4 FGDs; 26 participants)	Female	Mandera	6
			Wajir	6
		Male	Mandera	8
			Wajir	6
Semi-nomadic (8 FGDs; 65 participants)	Younger(4 FGDs; 31 participants)	Female	Wajir	6
			Mandera	9
		Male	Mandera	10
			Wajir	6
	Adult(4 FGDs; 34 participants)	Female	Wajir	6
			Mandera	10
		Male	Mandera	12
			Wajir	6
**Total**				16 FGDs; 122 participants

a. Younger age group: 18–25 years. Older age group: 26–49 years.

**Table 4.** Demographic characteristics of participants in individual interviews

	Female, *n* (%)	Male, *n* (%)	Total, *n* (%)
**Total**	24 (50)	24 (50)	48 (100)
**Age** 15–18 years 19–35 years 36–49 years	8 (33)8 (33)8 (33)	8 (33)8 (33)8 (33)	16 (33)16 (33)16 (33)
**Community type** Nomadic Semi-Nomadic	12 (50)12 (50)	12 (50)12 (50)	24 (50)24 (50)
**Marital status** Married Unmarried	20 (83)4 (17)	20 (83)4 (17)	40 (83)8 (17)
**Age married** 10–14 years 15–18 years 19–24 years 25–29 years	2 (10.5)15 (79)2 (10.5)0	05 (25)12 (60)3 (15)	2 (5)20 (51)14 (36)3 (8)
**Number of children One 2–5** 6–10 11+	2 (10)7 (35)11 (55)0	3 (17)4 (22)8 (44)3 (17)	5 (13)11 (29)19 (50)3 (8)

The results cover three overarching and related sub-themes: desired family size and fertility preferences, acceptable methods for child spacing, and reproductive decision makers. Within the first two themes, we present norms that emerged which supported large family sizes and sanctioned the use of modern FP for child spacing. The final theme presents findings on reproductive decision makers and the norm that assign this role to men.

### Desired family size and fertility preferences

Across focus groups and interviews, we identified a desire for large families as a strong social norm. FGD activities highlighted a group preference for eight or more children and thus a descriptive norm in support of larger families. Gender norms that women should bear many children and give birth immediately after marriage also contributed to a desire for a large family size. These norms were both descriptive (couples in the community complied with these) and injunctive (couples, and in particular women, who did not comply were sanctioned by community members who used derogatory terms against them). Taken together, these norms, alongside religious beliefs, explained the rationale behind large families. We first present the interplay between norms in support of large families, before presenting contextual religious factors that explain large family size.

#### It’s appropriate for women to give birth to many children

Participants, from both nomadic and semi-nomadic communities and independently of their age or sex, described a desire for large families. This ranged from eight to 15 children over the course of a woman’s lifetime and can be seen in a focus group participants’ reaction to the family size activity:

“*[F]ifteen is a good number, that is the maximum you have shown us and I think that is good for us [women] who are young and married, so that we can get as many children as possible before our time runs out*.” (Young woman 7, FGD-7, semi-nomadic)

Participants described large families as the norm. Group dynamics revealed an injunctive norm for large families (Table 1). For young nomadic men, this norm was particularly strong, as they stated the ideal family size to be up to and beyond 15 (higher than other groups). There was at times a disconnect between what FGD participants described as their ideal number and the “number of children achieved”. In three of the four FGDs with young female participants, overall, they anticipated having fewer children themselves than the ideal number, while adult groups described women in their communities to be achieving large families.

Women who give birth to children soon after marriage were seen as able to achieve large families. Having few children was unlucky and likened to having *one eye,* or as one female participant said in an adult focus group: “*one tree has no value, so we want many children*”. In addition, there were negative social sanctions for women and couples who had few children, including divorce, as a participant described of a woman who had four children:

“*After giving birth to only 4 children some people could say she is old that’s why she cannot have more children or your husband might not want you anymore and she could be stressed thinking that she could be divorced for this reason.*” (Older man, IDI-35, nomadic)

Desire for many children was particularly salient in FGDs, where participants described a desire for more children than they were currently having. One woman, for example, reflected on the difference between community fertility preferences and external pressures (from outside the community) to have fewer children.

“*We want many children. The government wants us to have few children, the children need food and they bring problems, but I still think the more children the better.*” (Adult female 4, FGD-4, semi-nomadic)

Having many children was viewed positively because children were able to help with household tasks and ensured the future of their parents, as seen in an FGD: “*What happens if our people get few children, who will the elders leave the animals to?*” (Young woman 3, FGD-8 semi-nomadic)

Some participants from nomadic communities also commented their lifestyles would be unsustainable without many children:

“*More children mean more blessings, you take one to school, another to Duksi [local Quranic school], another to herd the camels they will help you in some way later on in their lives.*” (Middle age female, IDI-3, nomadic)

This was also true in semi-nomadic settings, where children were viewed as essential to caring for animals:

“*I have goats, camel, cattle, donkeys and farm to farm. Who do you think will help me own and take care of all this? So, anything less than 8 is not desirable for a man to have as children.*” (Adult male 5, FGD-10, semi-nomadic)

A few participants invited the interviewer (and facilitator, in the case of FGDs) to reflect on their own norms and values, asking how many children the interviewers had. When interviewers described having few children, participants were shocked:

**Participant:** How many children do you have? **Interviewer**: One child […] **Participant**: Listen to that! How many more can you give birth to, you are now 28 and you have one child, you should be having 3 or 4 children, at the age of 40 you will not be giving birth, how many years do you have before you turn 40? (Adult female, IDI-3, nomadic)

Similarly, the interviewer presence may have in some instances influenced participant interactions during FGDs, with individuals thinking they should desire fewer children, as can be seen in an interaction between two adult men:

**Participant1**: I think four is very enough for a mother because more than 4 you can’t feed (*One of them shouts at him and tells them he is cursing his kids*) **Participant3:** *[shouts]* Yourself you have more than 10 why are you telling us 4 is a good number? (FGD-12, semi-nomadic)

Despite the pressure to have many children, and the associated benefits, some participants described economic and health consequences of having many children. For example, a young female FGD participant from a nomadic community said: “*I choose for people my age and myself to have a small family so that they can afford to educate them and have a good upbringing*”*.* This was reflected by a few others, from both nomadic and semi-nomadic communities, who felt fewer children was advantageous in the current economic climate:

“*I choose only three because it is easy to manage and raise them in the existing economy of the country. Also, the mother who gives birth to three will always look young and strong.*” (Adult male 8, FGD-11, semi-nomadic)

There were individual preferences for fewer children, despite descriptive and injunctive norms that supported larger families for both men and women.

#### Role of religion and fate

Religion and fate were often connected to discussions around family size. FGD and interview participants said that the number of children a woman had was determined by God, and religion was a reason for having many children. A nomadic adult female FGD participant described family size as “God’s plan”: “*All this is God’s plan; we are not all the same and so what God has planned for us is what we end up getting*” (Participant 6, FGD-1), *w*hile a participant in the younger male nomadic group described it as destiny: “*What [number of children] we will get is already destined by Allah, not us deciding, so ask another question please*” (Participant 7, FGD-14).

Alongside – and sometimes intertwined with – religion, fate also emerged in IDI narratives as an explanation for the number of children women ended up having, as a young woman described: “*Some of them [women] used family planning methods while for others, it is just fate*” (Young female, IDI-24, semi-nomadic). This was the case even when participants noted contexts that made it difficult for women to have children, as seen in a comment about a woman who had few children: “*What was she to do, her husband was never around? The problem was her husband. It was her fate*” (Middle age female, IDI-4, nomadic).

That children come from God at times deterred participants from being more direct about the expectation that women should have many children in both IDIs and FGDs. Participants described accepting whatever number of children they were given: “*It’s God who gives children*” (Adult female 2, FGD-1, semi-nomadic). Others echoed this, while still reflecting on the benefits of having many children.

“*Not that [a woman] hates what God has given her, but she would have wanted to take some of her children to the city, some to herd the animals … *” (Middle age male, IDI-28, nomadic)

Having many children was the norm, however, participants did not want to be ungrateful to God: “*[The community] are unhappy but not in bad faith. They feel sad and pray for her to get children*” (Older male, IDI-39, semi-nomadic). Similarly, during his interview a middle-aged man said of his nomadic community: “*They don’t say anything. It is God’s will*”. Similarly, a woman said: “*No one is unhappy, that is what God gave her*” (Middle age female, IDI-1, nomadic).

God provided children and community members prayed for more, as an older female participant described: “C*lose relatives and husbands sometimes wonder what happened to her and always pray to God to help her have other children*” (Older female, IDI-14, semi-nomadic). Religious beliefs explained fertility levels, but were also invoked to help community members and women who had fewer than the desired number of children process and explain their experiences.

### Acceptable methods for child spacing

While limiting children was not an option for community members (as large families were desired), both a descriptive and injunctive norm around healthy periods of child spacing emerged. Participants differentiated between two categories of spacing: using modern (or methods provided at a clinic) FP, and through breastfeeding. While child spacing of approximately two years was acceptable and encouraged through breastfeeding, there was an injunctive norm against the use of other methods of modern FP.

#### It’s appropriate for women to practice healthy child spacing

Child spacing was an important way for women and men to achieve their desired family size. As such they described that achieving spacing of two to three years was the norm, and had positive impacts on the health of women and children. Child spacing was viewed positively if a woman had already given birth to her first child; however, participants explained that different reasons and approaches used for child spacing were more or less acceptable.

Participants described that women should begin childbearing young, while maintaining birth spacing: “*[I]f she is above 18 I will tell her to give birth but space by three years*” (Adult male 2, FGD-11, semi-nomadic). Spacing often meant waiting until children were “grown” before having others. Participants discussed the benefits of spacing for the health of both the mother and child, as seen in a man’s comment: “*Child spacing is good because giving birth every year can lead to malnutrition and hence death*” (Adult male 2, FGD-9, nomadic). One woman described husbands accepted child spacing for health reasons:

“*[The husband] accepted it for the goodness of his children’s health and his wife’s health. If she is healthy the children will also be healthy. A difference of 3 years between children … *” (Older female, IDI-9, nomadic).

Spacing was achieved through various methods, including abstinence, breastfeeding, and a husbands’ absence due to herding or staying with another wife. One older female described child spacing as accepted if a husband had another wife to have children with: “*If [the husband] has another wife there is no problem*” (Participant 3, FGD-3, nomadic). For many, breastfeeding was described as common and typical as explained by a young woman’s comment: “*The only way to space your children is by breastfeeding, mostly 2 years*” (IDI-22, nomadic). Similarly, a male participant reflected on how this was commonly practised, yet it did not ensure long birth intervals: “*Women here just breastfeed, but there are women who give birth and immediately get pregnant again*” (Older male IDI-36, nomadic). Others described how breastfeeding prevented short birth intervals:

“*Mostly we breastfeed for long. This way, we do not get our monthly menstrual flow hence we do not get pregnant. We can stay up to 3 years, it is God’s plan.*” (Middle age female, IDI-8, semi-nomadic)

A few participants reflected that couples had little choice or control over how long currently practised methods allowed them to space for, despite a strong desire to space. This highlighted limited acceptable options for child spacing, other than periodic abstinence, being away for long periods of time or getting children from another wife: “*Unless [the husband] marries another wife, or goes away for years with the animals, there will be no other method*” (Young female 3, FGD7, semi-nomadic), and “*[To space] the husband will go to his second wife if he has another one*” (Adult male 1, FGD-9, semi-nomadic). Others mentioned breastfeeding as an acceptable method, but expressed a desire for additional methods to help spacing:

“*The way we know it is everyone should have a way to space her child. The child in the womb will suffer and the child who is already born will suffer. There is breastfeeding and a lot of hardship. If we knew it would be good.*” (Older female 1, FGD-1, semi-nomadic)

Comments from community members reflected an injunctive norm around child spacing, as healthy spacing periods were accepted and viewed positively (if achieved through the methods mentioned above). However, spacing for longer than two or three years was viewed negatively, as it limited the number of children a woman could give birth to. This is reflected in a focus group discussion comment, regarding the spacing period a husband might accept: “*If [child spacing] is only two years [the husband] might accept, if the spacing is longer he may not accept*” (Adult female 8, FGD-3, semi-nomadic). While women described spacing as important, comments highlighted that this had to be agreed to by the husband.

#### Women should not use modern methods of family planning

Child spacing through modern methods of FP was viewed as something used by “others” and this emerged across IDIs and FGDs. Modern methods of child spacing were described as going against community values, religious beliefs and were described as only used by “other” groups and not the research participants themselves. Most participants seemed to position themselves as knowing about modern FP, but suggested that in their rural areas (*baadia*) it was not used, as seen in a comment by one man: “*I have only heard of the injection used to space for 2 to 3 years, that’s all. They don’t use it in baadia*” (Middle age male, IDI-27, nomadic). Others explained that spacing periods of longer than two years were practised by those living in the city, and those who practised shorter spacing periods were judged, as described by an unmarried young male:

“*There is an injection and medication. Urban dwellers in the cities wait for up to 4 or 3 years before their next child, they even tell other people who wait for a year ‘Why do you give birth every other year? why don’t you space your children?*’” (Young male, IDI-48, semi-nomadic)

Participants provided examples where using modern FP was associated with negative social sanctions, including violence towards women and divorce, as stated by a male FGD participant’s comment: “*If [the husband] suspects [the wife] of anything to do with family planning, that is a clear sign of death*” (Adult male 8, FGD-9, nomadic). Only male FGD participants said FP use would end in divorce, however, female participants also described negative sanctions towards the couple: “*[T]he reputation of this family will be spoilt and everyone will keep speculating about their life*” (Adult female 6, FGD-4, semi-nomadic). Other participants also described gossip by community members*: “If their neighbours were to find out they could say that Halima and Said are no longer Muslims because they use these methods*” (Middle age male, IDI-28, nomadic).

There were judgments implicit in the rejection of modern methods, with some individuals being the exception to the rule. In these examples, certain women with *weak children* (a frequently repeated phrase in IDIs) and perceived to be unable to cope were seen as needing modern FP: “*Those women who bleed and have small weak children use [modern FP]*” (Older female, IDI-13, semi-nomadic). This also applied to sex workers: “*Pills and injections; I also heard about condoms but I have never seen it, they say it’s for prostitutes*” (Older female, IDI-5, semi-nomadic), as opposed to women deemed to be “inside” the community:

“*I will not go [to get FP], because family planning is against Islamic teachings, but people in rural area can go and get the services because they are struggling with many children.*” (Older man, IDI-37, nomadic)

Interestingly, when asked if they would visit local health workers providing modern FP, IDI responses were conflicting. For those who were already practising accepted child spacing periods, there was no need, however it was noted that others may need these methods:

“*I would not go, there is nothing I do not know. I have 12 children and they are spaced, why should I go? But for those who have small children and need to learn spacing, they can go and listen to them. As for me, I know how to go about it.*” (Older man, IDI-39, semi-nomadic)

“*My children are already spaced so why would I go to them? If someone gives birth with little space between their children might need it but my children are spaced.*” (Older female, IDI-12, nomadic)

While for others, there emerged a curiosity or willingness to access and learn about modern methods of child spacing, as seen in one comment: “*I will go and learn about it, it’s of good benefit to get new knowledge*” (Older man, IDI-33, nomadic). Participants placed emphasis on the importance of spacing and methods that permitted this:

“*We want it. We have small children and it’s very difficult to raise them, we need child spacing services a lot. It is good when children grow for some time before another one is born.*” (Middle age female, IDI-6, semi-nomadic)

For many participants, modern FP was assumed to be something that, alongside not being currently used, other community members (especially women) did not know about. This can be seen in an interaction between interviewer and participant, where the participant interrupts before the interviewer can ask about child spacing methods:

**Interviewer:** If a woman in this community wanted to use any child spacing methods … **Participant***: [interjects]* The women of this community don’t know such thing (Middle age man, IDI-27, nomadic)

Similarly, a young woman described community members did not use FP methods, hinting at a lack of education in her setting: “*They are not many [people using these methods]; many people here are not civilized people*” (Female, IDI-19, semi-nomadic). This tied in with an emerging narrative that community members believed modern FP may be too modern for certain groups.

### The role of men as reproductive decision makers

#### Men make reproductive decisions

Limiting children was often associated with using modern methods of FP to achieve child spacing, which was viewed negatively by community members and key reproductive health decision makers – mainly men.

Gender norms assigned husbands a decision-making role around child spacing. If women wanted to space children, they often required permission from their husbands first, as seen in an interview with a young man, from a nomadic community: “*She can talk to her husband and discuss it because he has to consent for her to do child spacing*”, and similarly in a focus group participant’s response to a vignette: “*She will tell her husband, because without his consent Saadia is not allowed to use family planning services*” (Adult male 2, FGD-12, semi-nomadic).

Men therefore played an important role in deciding the number of children a woman had. This was reflected in a comment by a man, regarding whether a woman could use FP methods or not: “*It all depends because it may be the woman has not attained the number of children the man wants*” (Adult male 4, FGD-10, semi-nomadic). When referring to how a husband would respond to a woman’s desire to use family planning methods if she had 10 children, a male participant described: “*The husband will accept her idea and say this number* [10] *is already enough for one woman*” (Young male 1, FGD-15, semi-nomadic). Men’s role and dominance were particularly relevant when women had not achieved the desired number of children or given birth to a son, as seen in a young male’s comment about the husband of a woman who had three daughters: “*It is not that he did not want daughters but he felt like they were too many since he had no son, he wanted a son*” (Young male, IDI-48, semi-nomadic). Discussions around spacing were often not permitted until couples had a son. This was seen in a man’s comment, where he described the use of violence to assert his role as decision maker: “*The husband will even strangle [the wife], he can’t accept. You cannot give birth to four girls and tell me you want to space. Ladies are like feathers; you do what you want with them*” (Adult male 7, FGD9, nomadic). Another FGD participant described a couple “*will fight and divorce will be next*”, if a woman is found to be discussing FP.

## Discussion

Based on interviews and FGDs with nomadic and semi-nomadic pastoralists in North Eastern Kenya, our findings provide insights into reproductive health preferences from a community perspective. This paper explores themes around desired family size and fertility preferences, acceptable methods for child spacing, and reproductive decision making. We find norms supporting large family size and child spacing, while at the same time norms that sanction modern FP use. Gender norms assigned men reproductive decision-making powers, which means they often have the final say around the number of children a woman has and child spacing periods. Our findings also uncover key tensions between community norms which support large family sizes and address overall health needs, and the aims of global FP programmes, which promote the use of modern FP for healthy timing and spacing of pregnancy. This tension echoes other literature on FP preferences, which highlights how differences in values may affect the acceptability of FP.^52^ This study highlights the need for targeted research on social norms and community preferences. Findings from this study can inform context-specific FP interventions, as well as broader health programmes, in North East Kenya.

We find large families are important for both semi-nomadic and nomadic women and men, echoing other research in the region.^28^ For the majority of participants, large families were the norm; this was both common (a descriptive norm held by other community members) and acceptable (an injunctive norm linked to sanctions). As such, this norm was strong (obligatory) and formed part of a collective purpose.^23,53^ FGD participants described their desire for large families, and this norm was reinforced and amplified in group settings. Couples (and women in particular) who had few children, faced negative consequences and sanctions, including judgment from others in the community. Participants also described the social and practical value of having many children, and some challenged FP programmes that seek to reduce this number. Few participants described the health and economic benefits of having relatively smaller families and fewer still believed this decision would be met with community support. However, while the sample is small, younger female FGD participants expected to have fewer children than the number typically achieved in their community. This is possibly indicative of norm change, towards smaller families amongst this group, and suggests the need for further research. This was not the case for younger male groups. A small number of participants, across all age groups, described that smaller families were appropriate. This echoes what we found in our separate analysis on early marriage.^45^ Some individuals expressed a desire to have smaller families, which may reveal shifts in perceptions and norms.

Our findings illustrate the importance of religion and fate in sustaining preferences for large families, at times also explaining smaller family size. Religion and fate are often viewed as intertwined and as external, almost-omniscient forces or powers. Similar to other studies,^28,29^ only God was responsible for how many children a woman had, which may explain that at times there was reticence to express dissatisfaction with smaller family sizes; to do so may demonstrate a lack of faith. For some, attributing family size to God and/or fate may be a more acceptable way of understanding limitations to preferred family size, making it something that is bestowed rather than resulting from decisions made by women and men themselves. How family size was conceptualised may have made it hard for participants to accept modern FP as offering opportunities for choice and decision-making, where choice may in fact be counter to community values and norms. This finding has implications for programming focused on increasing FP knowledge and access as a means of empowering individuals to make informed decisions. Continued work may be needed to closely implicate cultural and religious leaders in designing and implementing SBC strategies that drive interventions, focused on an Islamic understanding of child spacing (which is permitted within the confines of marriage), throughout the project cycle.^54^

Child spacing emerged as a way to achieve desired family size and different patterns and judgments around its acceptability emerged. Seemingly in tension with the norm for large families, child spacing of between two and three years was actively encouraged. Participants pointed to their own spaced families, describing how others should also space. Mostly, child spacing was practised for the health of the mother and child, through both breastfeeding and abstinence methods. This indicates a positive norm, defining an appropriate behaviour, around spacing that has been leveraged as an entry point for FP programmes elsewhere,^17–20^ and could be leveraged for future interventions among nomadic and semi-nomadic communities.

The use of other modern methods for child spacing carried sanctions and different kinds of judgments (revealing an injunctive norm, where individuals could anticipate negative sanctions from community members). This is similar to research on FP sanctions in other settings.^55,56^ These may be tied to gender norms where women are expected to produce many children, whereas women who are seen as not able to cope with having children (“women who bleed”) and sex workers are seen as the anomalies who access modern FP. The judgments inherent in IDI participants’ statements about modern FP use suggest that there is stigma associated with using modern FP. The perception that modern FP is for “others” (e.g. urban populations, civilised people) but not the research participants themselves or women “inside” the community, suggests more work is needed to normalise the use of modern contraception beyond outlier users. Our findings underscore the importance of FP programs that engage with desired family sizes and existing methods for child spacing. In particular, gender norms that assign men reproductive decision-making power in this context, as has been identified in our other work,^46^ also play a role in access to FP for women in these communities. Despite this, fewer women in the North East of Kenya report that their husband knows they are using a method of FP,^3^ compared with the national average. Men’s role in making reproductive decisions has implications for programmatic interventions, requiring tailored, gender-responsive SBC strategies that include cultural, religious, and community leaders.^49^

Some participants in this study drew attention to the clashes between their own values and external values from other populations living in urban settings, the government, and the health system about having fewer children. This is particularly pertinent in a setting where nomadic and semi-nomadic groups have historically been viewed as different, marginalised from state services and/or pushed to settle. The contrast in values even emerged in how research participants and interviewers interacted. In a reversal of the typical research scenario, at times interviewers were asked about their own family size and participants expressed judgment about how interviewers cared for their children and declined to answer questions about family size. The disconnect in ideologies and norms between the interviewers and participants illustrates broader challenges faced by those who work in public health, particularly around how certain groups hold power to define and enforce values upon others. This dynamic may have been present in interviews, such that participants may have assumed interviewers held particular views associated with international FP agendas. This is relevant because prior research shows low uptake of FP can result from programmes being viewed as “colonial” and “imperialist”.^57^ From the perspective of nomadic and semi-nomadic communities, it may be that as well as interventions seeking to change family size, the research process itself is viewed as carrying value judgments about pastoralist communities. This has implications for how local and international agencies communicate with communities, design and choose research methods, conduct research, and train their enumerators/staff. Further research, conducted in collaboration with nomadic and semi-nomadic communities, has been shown to be effective and will be instructive in designing approaches to engaging with and changing gender and social norms related to FP.^58^

Our paper has three main limitations. First, while interviewers were from Wajir and Mandera counties (and spoke the local languages), they were not from the nomadic and semi-nomadic communities interviewed. As such, they may have been viewed as outsiders by the participants, affecting social desirability, such as comments relating to preferences for smaller families and/or use of modern FP. Second, interview dynamics may have been influenced by differences in nomadic status and education level between interviewers and participants. To mitigate this, interviewers had training on conducting ethical qualitative research on sensitive topics prior to data collection, where power dynamics were explored, and interviews were conducted with same sex interviewers. Daily debriefs with the whole research team were carried out, which allowed us to identify any challenges and respond to these if they came up. Thirdly, this research is exploratory in nature and the relatively small sample size means findings may not be generalisable to other nomadic and semi-nomadic communities in North Eastern Kenya. Relatedly, we were unable to analyse in detail norm change across age groups. We analysed qualitative data across FGDs and IDIs to validate our findings, and to better explore the complex relationships between norms, fertility preferences, and family planning, however, the findings may be limited to these populations.

## Conclusion

Family planning research and interventions must take a community- and women-centred approach, through collaboratively exploring norms, religion, and lifestyle factors that surround FP, if they are to succeed in ensuring equitable and rights-based access to FP. Fertility preferences play a key role in explaining low uptake of FP. However, there exist seemingly contradictory norms around family size and length of child spacing, while gender norms limit women’s reproductive choice. Further research is needed among nomadic populations, exploring the role of religion and fate in sustaining FP norms; how these factors may preclude individual decision-making on FP and child spacing is especially needed. The perceived benefits of FP for women’s and children’s health are an entry point for interventions looking to increase awareness and access to modern FP, while considering that nomadic and semi-nomadic women and men have their own preferred methods of spacing and are wary of “foreign” interventions. Intentionally co-creating SBC strategies with pastoralist communities early on is useful to ensuring these preferences and concerns are not only documented but integrally woven into the design, implementation, and evaluation of the intervention.
